# Clinical evaluation of serum miR-513a-3p combined with arterial blood gas analysis parameters and lung ultrasound score in neonatal respiratory distress syndrome

**DOI:** 10.1186/s13052-024-01795-7

**Published:** 2024-10-29

**Authors:** Tingting Du, Hui Lei, Jian Dong, Ye Wang, Jun Li

**Affiliations:** 1 Department of Ultrasound Medicine, The First Affiliated Hospital of Shihezi University, No. 107, Community 32, North Second Road, Shihezi, 832008 China; 2 Department of Neurosurgery, Hui Autonomous Prefecture People’s Hospital, Changji, 831100 China

**Keywords:** NRDS, miR-513a-3p, Diagnosis, Prognosis

## Abstract

**Background:**

Neonatal respiratory distress syndrome (NRDS) is harmful to neonates and the prognosis is variable, ranging from mild to severe forms. This study aims to evaluate the clinical utility of miR-513a-3p in conjunction with arterial blood gas analysis parameters and lung ultrasound (LUS) score in the context of NRDS.

**Methods:**

The study included 169 preterm infants, including 106 newborns with NRDS and 63 newborns without NRDS. The relative expression level of miR-513a-3p was detected by quantitative real time polymerase chain reaction (qRT-PCR). Umbilical artery blood gas parameter values and LUS score were recorded, and the clinical significance of miR-513a-3p, umbilical artery blood gas parameter and LUS score in NRDS were evaluated by Receiver Operating Characteristic (ROC) analysis.

**Results:**

Elevated levels of miR-513a-3p were detected in the serum of NRDS, and higher expression of miR-513a-3p was observed in individuals with poor prognosis. Notably, miR-513a-3p exhibited a significant correlation with the parameters of arterial blood gas analysis and LUS score in NRDS patients. Furthermore, miR-513a-3p was one of the risk factors for poor prognosis in NRDS patients. miR-513a-3p levels combined with umbilical artery blood gas parameters and LUS score has diagnostic value for NRDS and is reliable for its prognosis.

**Conclusions:**

Elevated levels of miR-513a-3p in neonatal serum served as a useful tool in the combined assessment with umbilical artery blood gas analysis and LUS score to diagnosis and prognosis of NRDS. Consequently, miR-513a-3p may be served as a biomarker for diagnosis and prognosis of NRDS.

## Background

Neonatal respiratory distress syndrome (NRDS), also known as neonatal pulmonary hyaluronosis, encompasses detrimental symptoms like escalating breathlessness and respiratory insufficiency that arise promptly following delivery [1]. The presenting clinical signs encompass irregularities in respiratory rate, the production of grunting sounds, evident retraction of the intercostal spaces, and cyanosis, characterized by a bluish discoloration of the skin [2]. Additional supportive treatments for NRDS are essential to alleviate the severity of NRDS and mitigate associated complications. This is particularly important as the incidence of NRDS rises due to the increasing number of preterm births, which is a consequence of advancements in obstetric care [3–5].

In general, the pH balance within newborn infants is stable. When a fetus experiences hypoxia, anaerobic respiration takes over as the primary method for generating energy. This metabolic switch leads to the production of numerous acidic by-products, which may ultimately result in damage to the organs of the infant [6, 7]. Umbilical arterial blood gas analysis is widely recognized as a reliable tool of fetal and neonatal ischemia and hypoxia. It provides an objective assessment of the metabolic condition of the tissues and organs [8].

The examination of neonatal lung ultrasound (LUS) is garnering considerable and increasing attention within clinical research, yielding valuable clinical applications [9]. LUS constitutes a point-of-care, user-friendly, non-ionizing, immediate, and reliable diagnostic method that can be readily repeated. The indicators of LUS exhibit minimal variation with advancing age, making it particularly well-suited for application in neonates and in the intensive care unit (ICU) environment [10]. LUS functions as a potent diagnostic method and a non-invasive investigative instrument for the qualitative assessment of neonatal respiratory conditions [9].

Alterations in the expression patterns of microRNAs (miRNAs) have been pivotal in identifying the stages of disease onset and progression, thereby establishing a critical foundation for discovering novel and effective biomarkers in clinical settings [11]. Recent research consistently underscores the role of of miRNA in the advancement of respiratory distress [12–14]. Notably, the aberrant expression of miR-513a-3p has been documented in both blood and bronchoalveolar fluid samples from individuals suffering from acute respiratory distress syndrome [15]. In studies involving preterm infants experiencing respiratory distress, an increased expression of miR-513a-3p was observed [16]. However, the clinical application of miR-513a-3p in NRDS has not been studied.

## Methods

### Research subjects and clinical data collection

The present investigation received ethical clearance from the Research Ethics Board at The First Affiliated Hospital of Shihezi University, Xinjiang, China, all children’s legal guardians were informed and voluntarily signed the informed consent form. The study encompassed a sample of 169 preterm infants delivered at The First Affiliated Hospital of Shihezi University from 2019 to 2023, including 106 cases diagnosed with NRDS and an additional 63 healthy control subjects. The average gestational age for the control group and the NRDS group were 31.97 ± 2.28 weeks and 31.77 ± 2.25 weeks, respectively. The diagnosis of NRDS was primarily based on clinical manifestations and imaging results, characterized by a respiratory rate exceeding 60 breaths per minute, labored breathing, respiratory distress, cyanosis, and chest retractions in the intercostal or subcostal areas, or above the sternum. Additionally, imaging revealed bronchial obstruction, reticulated patterns, or areas of opacity in the lungs on X-ray examination. Patients harboring primary insufficiencies of alveolar surfactant, genetic congenital heart defects, preexisting metabolic irregularities, and anomalies of the pulmonary and thoracic walls were not included in the study. To exclude the influence of genetic conditions, we also referred to previous studies [17–23].

Basic data were collected, including gender, birth weight, gestational age, and blood gas oxygenation index. The basic data of the control group and the case group are shown in Table 1. There is no difference in gender, delivery weight and gestational age of newborns between the two groups, and the blood gas oxygenation index of patients in the NRDS group is significantly lower than that in the control group.

**Table 1:** Basic information statistics of subjects

Parameters	Control (*n* = 63)	NRDS (*n* = 106)	*p*-value
Gender (male/female)	28/35	56/50	0.292
Gestational week of delivery (weeks)	31.97 ± 2.28	31.77 ± 2.25	0.569
Birth weight (g)	2913.16 ± 198.16	2858.42 ± 165.22	0.055
Blood gas oxygenation index (mmHg)	174.21 ± 8.99	132.58 ± 27.20	<0.001

All data were presented as mean ± standard deviation

### Neonatal umbilical artery blood gas analysis

After birth and before the newborn’s first cry, two hemostatic clamps were used to clamp the proximal umbilical cord of the fetus, about 15 cm from the fetus’s navel, to block the blood circulation between the newborn and the placenta. Umbilical artery blood was collected using a heparinized syringe connected to the scalp needle. The umbilical artery blood gas of the newborn was examined by blood gas analyzer. The indicators include pH (Pondus Hydrogenii), PaO_2_ (Arterial Oxygen Partial Pressure), PaCO_2_ (Arterial Carbon Dioxide Partial Pressure), HCO_3_^−^ (bicarbonate), and BE (Bicarbonate Excess). The whole test procedure should be completed within 30 min to preserve the integrity of the active components present in the newborn’s umbilical artery blood, thereby ensuring the accuracy of the test results.

### Pulmonary ultrasonography

Each side of the lung was divided into anterior, lateral and posterior regions by parasternal line, anterior axillary line, posterior axillary line and posterior median line. Then, each lung is divided into two upper and lower regions by the middle line of the two nipples, making a total of 12 regions. The intercostal spaces were examined from the second intercostal space from the top to the bottom and from the inside to the outside with a line-array probe, followed by a parallel examination of each of the 12 regions of the lungs bilaterally, and the sonograms of each region of each side of the lung were stored and recorded. The scoring criteria refer to the criteria proposed by Rouby [24] and Sartori [25] et al. LUS score in the 12 regions of double lungs were the sum of the 12 regions, and the score ranged from 0 to 48 points.

### Quantitative real-time PCR (qRT-PCR)

Neonatal venous blood was taken and centrifuged at a low temperature, then serum was collected. Total RNA was recovered from the blood serum using the TRIzol reagent (Invitrogen; Thermo Fisher Scientific, Waltham, MA, USA). The quality and quantity of the isolated RNA were assessed using a NanoDrop 2000 spectrophotometer (Invitrogen; Thermo Fisher Scientific, Waltham, MA, USA) in accordance with the manufacturer’s protocol. Upon achieving an optical density ratio of A260/280 approximately equal to 2.0, the RNA was chosen for additional analysis. The isolated RNA was converted into complementary DNA (cDNA) through reverse transcription with the PrimeScript RT Reagent Kit (TaKaRa; Japan). The SYBR Green I Master Mix kit (Invitrogen; Thermo Fisher Scientific, Waltham, MA, USA) was employed for qRT-PCR to determine the comparative expression levels of miR-513a-3p. The sequences of the primers were as follows:

miR513a-3p forward: 5’-TAAATTTCACCTTTCTGAGAAGG-3’,

miR-513a-3p reverse: 5’-GCGAGCACAGAATTAATACGAC-3’.

GAPDH forward: 5’-GGGAAACTGTGGCGTGAT-3’.

GAPDH reverse: 5’-GAGTGGGTGTCGCTGTTGA-3’.

### Records of prognostic outcomes in the experimental group

The therapeutic outcomes at the 28-day mark were meticulously documented and categorized into two distinct categories. Favorable resolution or recovery: subsequent to the intervention, the neonate exhibited stable vital parameters, a notable reduction or complete resolution of respiratory disturbances, and a nearly standard chest radiograph. Consequently, the neonate was able to be weaned off mechanical ventilation and supplemental oxygen. Abandonment or death: therapeutic efforts were deemed unsuccessful, leading to persisting instability in the neonate’s vital signs. The neonate remained dependent on mechanical ventilation, encountered grave complications, or had treatment withdrawal requested by relatives. Consequently, the neonate was discharged from the healthcare facility or vital signs ceased, ultimately resulting in a clinical determination of death, often due to economic constraints or poor prognosis.

### Statistical analysis

SPSS 26.0 and GraphPad Prism 9 were used for data processing and statistical evaluation. Data were expressed as mean ± standard deviation (SD) and independent sample t test was used. The logistic regression analysis was used to analyze the risk factors of poor prognosis in NRDS patients. ROC curve was used to evaluate the diagnostic ability of miR-513a-3p, arterial blood gas parameters and LUS score on NRDS and the predictive effect of prognosis. Pearson analysis was used for correlation analysis, and a p-value less than 0.05 was considered statistically significant.

## Results

### Differential expression of miR-513a-3p

**Fig. 1:**
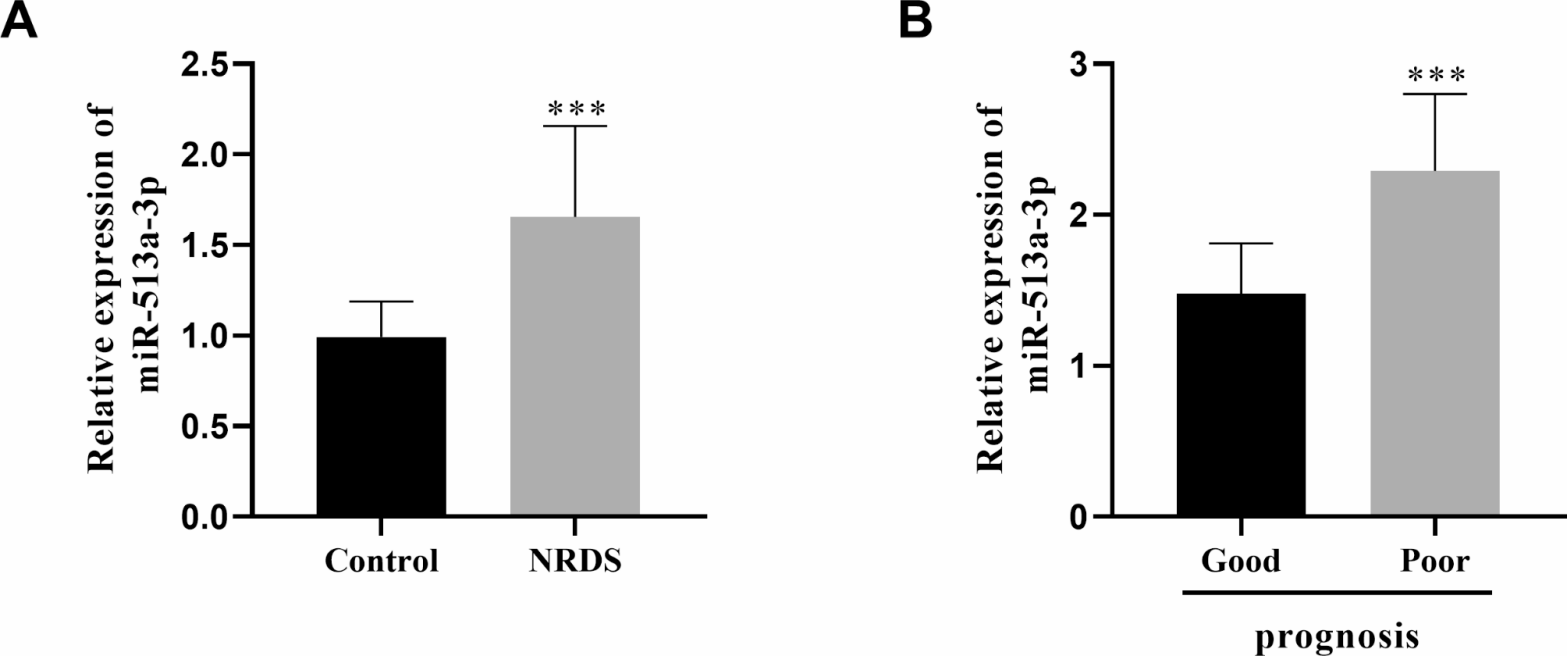
Differential expression of miR-513a-3p. (**A**) The expression of miR-513a-3p in NRDS was detected by qRT-PCR (**B**) The expression of miR-513a-3p was detected by qRT-PCR in patients with different prognostic conditions. ^***^P<0.001

The serum expression level of miR-513a-3p was significantly elevated in NRDS patients compared to the control group (*p* < 0.001, Fig. 1A). Depending on the treatment outcome, 83 infants had a good prognosis and 23 infants had a poor prognosis. We examined the varying expression patterns of miR-513a-3p in infants with different prognosis. As depicted in Fig. 1B, a higher expression of miR-513a-3p was detected in those infants with a poor prognosis (*p* < 0.001).

### Comparison of arterial blood gas analysis parameters and LUS score

As shown in Table 2, regarding the blood gas parameters of the umbilical artery, the analysis revealed that the pH level, PaO_2_, HCO_3_^−^ concentration, and BE value were all reduced in NRDS compared to the control group. Conversely, the PaCO_2_ in the NRDS group was observed to be higher than that of the control group (*p* < 0.001). The mean LUS score across the 12 zones for the control group was 24.52 ± 8.63, whereas for the infants with NRDS, the score was 38.06 ± 8.21. The infants with NRDS exhibited significantly higher scores than those without NRDS (*p* < 0.001).

**Table 2:** Comparison of arterial blood gas analysis parameters and LUS score between control group and NRDS group

Parameters	Control (*n* = 63)	NRDS (*n* = 106)	*p*-value
Arterial blood gas analysis index			
pH	7.36 ± 0.06	7.27 ± 0.06	<0.001
PaCO_2_(mmHg)	44.33 ± 6.87	56.96 ± 9.69	<0.001
PaO_2_(mmHg)	74.55 ± 5.69	63.76 ± 6.15	<0.001
HCO_3_^−^ (mmol/L)	24.49 ± 3.62	16.74 ± 4.41	<0.001
BE (mmol/L)	-6.07 ± 1.80	-8.70 ± 1.97	<0.001
LUS score (points)	24.52 ± 8.63	38.06 ± 8.21	<0.001

LUS, Lung ultrasound; pH, Pondus Hydrogenii; PaCO_2_, Arterial Carbon Dioxide Partial Pressure; PaO_2_, Arterial Oxygen Partial Pressure; BE, Bicarbonate Excess. All data were presented as mean ± standard deviation

Moreover, we conducted a comparison of the arterial blood gas analysis parameters and the LUS score among patients with varying prognosis (Table 3). The pH value (*p* < 0.001), PaO_2_ (*p* < 0.001), HCO_3_^−^ concentration (*p* = 0.018) and BE value (*p* < 0.001) of umbilical arterial blood of infants with poor prognosis were lower than those of infants with good prognosis. Infants with poor prognosis had high PaCO_2_ in umbilical arterial blood (*p* = 0.007). Notably, the LUS score in patients with a poor prognosis was substantially higher at 42.74 ± 7.34, in contrast to those with a favorable prognosis, who had a LUS score of 36.76 ± 8.00 (*p* = 0.002).

**Table 3:** Comparison of arterial blood gas analysis parameters and LUS score in patients with different prognosis

Parameters	Prognosis		*p*-value
	Good (*n* = 83)	Poor (*n* = 23)	
Arterial blood gas analysis index			
pH	7.29 ± 0.05	7.22 ± 0.05	<0.001
PaCO_2_ (mmHg)	55.63 ± 9.10	61.75 ± 10.43	0.007
PaO_2_ (mmHg)	65.01 ± 5.20	59.24 ± 7.27	<0.001
HCO_3_^−^ (mmol/L)	17.27 ± 4.46	16.33 ± 3.74	0.018
BE (mmol/L)	-8.25 ± 1.69	-10.30 ± 2.10	<0.001
LUS score (points)	36.76 ± 8.00	42.74 ± 7.34	0.002

LUS, Lung ultrasound; pH, Pondus Hydrogenii; PaCO_2_, Arterial Carbon Dioxide Partial Pressure; PaO_2_, Arterial Oxygen Partial Pressure; BE, Bicarbonate Excess. All data were presented as mean ± standard deviation

### Correlation of arterial blood gas analysis parameters and LUS score with miR-513a-3p in NRDS patients

To investigate the relationship between arterial blood gas analysis parameters, LUS score, and serum levels of miR-513a-3p in NRDS, a Pearson correlation analysis was conducted. Table 4 illustrated that arterial pH, PaO_2_, HCO_3_^−^ level, and BE level exhibited a negative correlation with the expression levels of miR-513a-3p. On the other hand, both PaCO_2_ and LUS score were positively correlated with miR-513a-3p expression.

**Table 4:** Correlation of arterial blood gas analysis parameters and LUS scores with miR-513a-3p in NRDS patients

Parameters	Correlation with miR-513a-3p (*r*)	*p*-value
Arterial blood gas analysis index		
pH	-0.6319	<0.001
PaCO_2_	0.5933	<0.001
PaO_2_	-0.5844	<0.001
HCO_3_^−^	-0.5234	<0.001
BE	-0.6079	<0.001
LUS score	0.6872	<0.001

LUS, Lung ultrasound; pH, Pondus Hydrogenii; PaCO_2_, Arterial Carbon Dioxide Partial Pressure; PaO_2_, Arterial Oxygen Partial Pressure; BE, Bicarbonate Excess

### The level of miR-513a-3p is a risk factor for poor prognosis in NRDS patients

miR-513a-3p levels, arterial blood gas parameters and LUS score were included in logistic regression analysis according to the prognosis of NRDS patients. The results showed that miR-513a-5p emerged as a significant risk factor associated with poor prognosis in NRDS patients (Table 5).

**Table 5:** Logistic regression analysis of risk factors for poor prognosis in patients with NRDS

Parameters	Standard error	OR	95%CI	*p*-value
miR-315a-3p	0.803	0.103	0.021–0.496	0.005
pH	0.802	0.113	0.023–0.543	0.007
PaCO2	1.016	0.316	0.043–2.319	0.257
PaO2	0.842	0.686	0.132–3.569	0.654
HCO3-	0.778	0.445	0.097–2.047	0.299
BE	0.984	0.323	0.047–2.221	0.251
LUS score	0.912	0.152	0.025–0.908	0.039

LUS, Lung ultrasound; pH, Pondus Hydrogenii; PaCO_2_, Arterial Carbon Dioxide Partial Pressure; PaO_2_, Arterial Oxygen Partial Pressure; BE, Bicarbonate Excess

### Clinical value of miR-513a-3p, LUS score and arterial blood gas parameters in NRDS

**Fig. 2:**
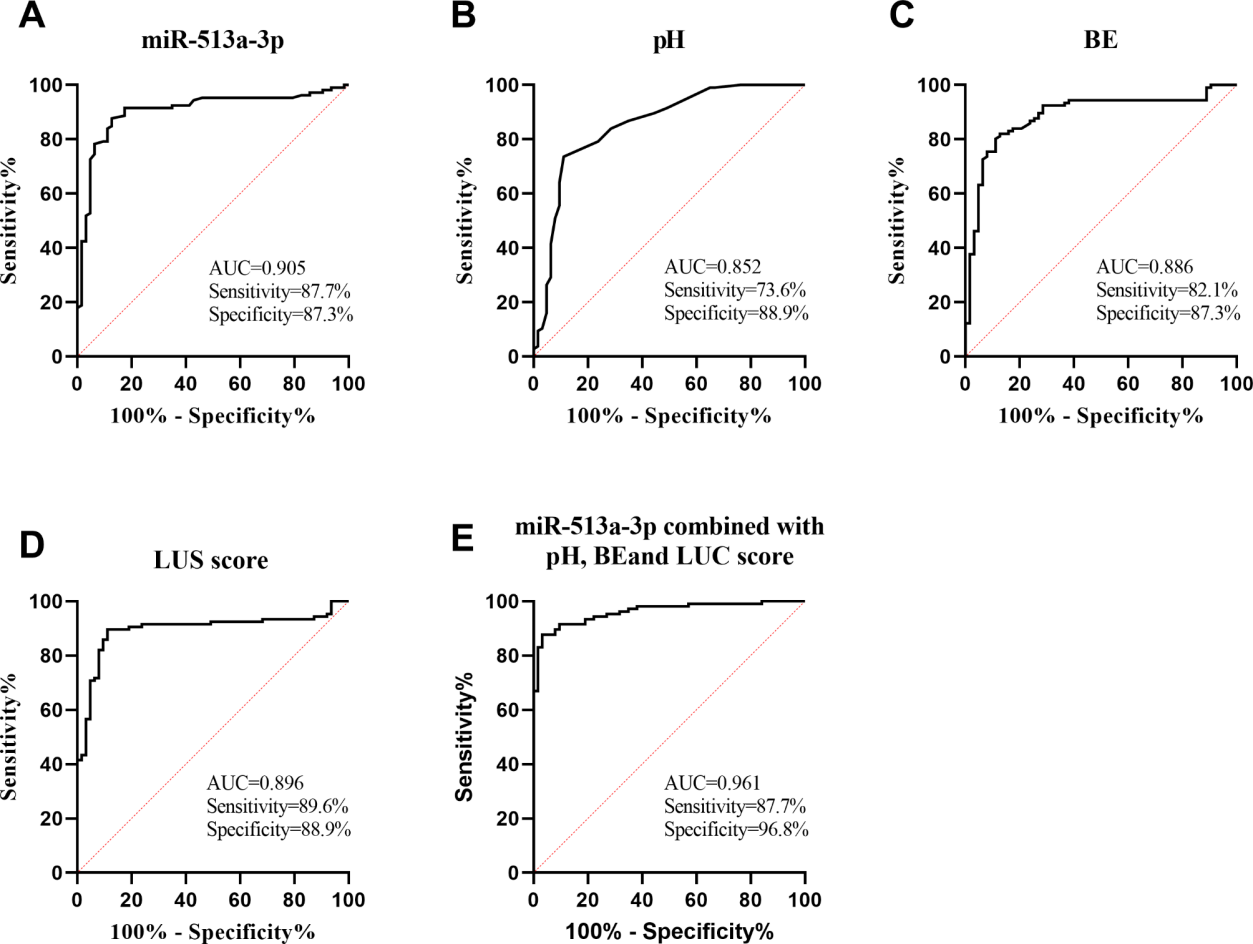
ROC curve of miR-513a-3p and arterial blood gas parameters and LUS score in diagnosis of NRDS. (**A**) ROC curve of miR-513a-3p for NRDS diagnosis. (**B**-**C**) ROC curve of umbilical arterial blood pH value and BE value in diagnosis of NRDS. (**D**) ROC curve of LUS score for NRDS diagnosis. (**E**) ROC curve of miR-513a-3p combined with arterial blood gas parameters and LUS score for NRDS diagnosis

To assess the diagnostic efficacy of miR-513a-3p, arterial blood gas analysis parameters and LUS score in NRDS, ROC analysis was conducted. As presented in Fig. 2A, the ROC analysis of miR-513a-3p for NRDS diagnosis yielded an AUC of 0.905. The AUC for pH and BE values of umbilical arterial blood in diagnosing NRDS were 0.852 and 0.886, respectively (Fig. 2B and C). For LUS score, its AUC in diagnosing NRDS was 0.896 (Fig. 2D). miR-513a-3p combined with arterial blood gas parameters and LUS score had higher diagnostic value for NRDS (Fig. 2E).

**Fig. 3:**
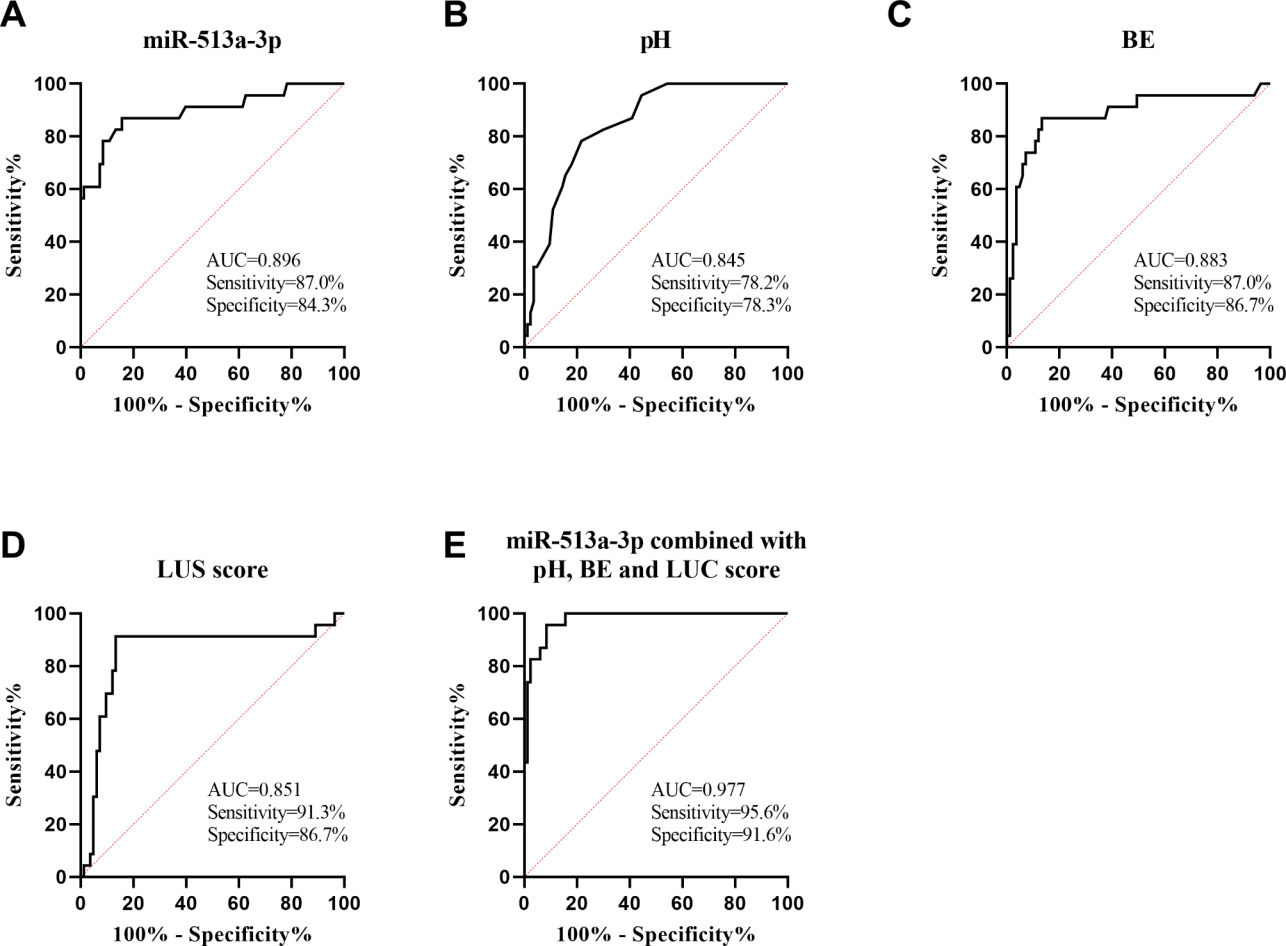
ROC curve of miR-513a-3p and arterial blood gas parameters and LUS score predicting NRDS prognosis. (**A**) ROC curve of miR-513a-3p in predicting NRDS prognosis. (**B**-**C**) ROC curve of umbilical arterial blood pH value and BE value in predicting NRDS prognosis. (**D**) ROC curve of LUS score in predicting NRDS prognosis. (**E**) ROC curve of miR-513a-3p combined with arterial blood gas parameters and LUS score predicting NRDS prognosis

The results showed that miR-513a-3p level, umbilical artery blood pH value, BE value and LUS score for predicting neonatal prognosis of NRDS was 0.896, 0.845, 0.883, 0.851, respectively (Fig. 3A and D). The integrated influence of these variables enhanced the value of predicting the outcome of NRDS, yielding an AUC of 0.977 (Fig. 3E).

## Discussion

The majority of infants diagnosed with NRDS are born preterm [26]. The prevalence of these cases correlates with gestational age, which tends to rise as birth weights are lower than normal for the gestational age [27]. Accurate early identification of the likelihood of NRDS and proactive measures for its prevention and management are crucial for enhancing the outcomes of premature infants.

The significance of umbilical artery blood gas analysis in neonates in obstetrics has been increasingly recognized. As demonstrated in Patil ‘s research, alterations in the blood gas parameters of the umbilical artery are associated with the incidence of asphyxia. A decrease in pH value, PaO_2_, or an increase in PaCO_2_ levels indicates a heightened risk of neonatal asphyxia, with a more severe manifestation corresponding to greater deviations from normal blood gas parameters [28]. Arterial blood gas analysis serves as a crucial tool for medical professionals in the delivery suite, facilitating the early identification of neonates vulnerable to respiratory distress syndrome and enhancing the postpartum care provided to these infants [29].

The utility of the LUS score for NRDS has been validated through numerous research endeavors. For instance, research has shown that LUS score is dependable in the evaluation of NRDS. The LUS score varied across the groups classified as non-NRDS, mild NRDS, and severe NRDS [30]. In a study led by Houqing Pang and colleagues, the LUS score along with the areas of consolidation are capable of differentiating NRDS from non-NRDS cases and identifying the varying stages of NRDS, while also acting as a prognosticator for the necessity of mechanical ventilation [31].

It was found that the pH level, PaO_2_, HCO_3_^−^ concentration, and BE were all lower in neonates with NRDS, in contrast to the control group. On the other hand, the LUS score and PaCO_2_ in the NRDS group was noted to be greater. These indicators also showed the same trend in children with poor prognosis compared to those with a good prognosis. These results suggested that patients with NRDS have a relatively high risk of asphyxia, poor lung condition, and less than ideal ventilation status, and it is more severe in patients with poor prognosis.

Certain studies have further established a strong correlation between miRNAs and NRDS, for instance, elevated levels of miR-375 have been linked to the development of NRDS and its clinical outcomes in neonates [32]. Our finding indicated that the expression of miR-513a-3p was increased in neonates with NRDS, and this upregulation was more pronounced in those with a poorer prognosis. This finding aligns with prior scholarly inquiries [16]. In order to further explore the relationship between the elevation of miR-513a-3p and the disease status of patients, we conducted correlation analysis between miR-513a-3p and arterial blood gas parameter values and LUS score. As anticipated, the expression of miR-513a-3p demonstrated significant correlations with clinical indicators, suggesting that higher levels of miR-513a-3p may correlate with increased symptom severity in patients. Although a large number of studies have confirmed that arterial blood gas parameters and LUS have a good diagnostic effect on NRDS, the detection of serum miR-513a-5p levels is relatively convenient and more maneuverable. More importantly, miR-513a-3p also plays a significant role in the prognosis of NRDS. Meanwhile, logistic regression analysis further indicated that miR-513a-3p was one of the risk factors for poor prognosis in NRDS patients. These results suggested that miR-513a-3p may be one of the biomarkers for NRDS patients to be used in the daily care.

Previous studies have reported that effective perinatal and neonatal care could substantially reduce fetal mortality or morbidity [33, 34]. Due to the abnormal expression of miR-513a-3p in the serum of NRDS infants, it is imperative to explore whether the expression of miR-513a-3p is similarly altered in in the blood of the mothers of these infants, or whether the expression of miR-513a-3p can also predict the occurrence of NRDS before the birth of the fetus or before the emergence of clinical manifestations.

In this study, we mainly explored the clinical value of serum miR-513a-3p combined with arterial blood gas analysis parameters and LUS score in NRDS. Nonetheless, the limitation of our study was not providing insight into the precise mechanism through which miR-513a-3p influences NRDS. In addition, we did not assess the clinical Silverman score to determine the severity of NRDS, which is another weakness of our study. Moreover, the evaluation of the results may be affected by the number and population characteristics of the studied samples, thus, a larger sample size may be more persuasive for the findings.

## Conclusion

This investigation revealed that the levels of miR-513a-3p in the blood serum are elevated among individuals experiencing NRDS. Furthermore, those with a less favorable prognosis exhibited significantly higher serum miR-513a-3p expression compared to those with a positive outcome. The miR-513a-3p expression, along with arterial blood gas analysis metrics and LUS score, serves as a valuable diagnostic tool for NRDS and exhibits considerable predictive capabilities for patient prognosis.

## Data Availability

The datasets used and/or analysed during the current study are available from the corresponding author on reasonable request.

## Abbreviations

BE: Bicarbonate Excess
cDNA: Complementary DNA
HCO_3_^−^: Bicarbonate
ICU: Intensive Care Unit
LUS: Lung Ultrasound
miRNAs: MicroRNAs
NRDS: Neonatal respiratory distress syndrome
PaCO_2_: Arterial Carbon Dioxide Partial Pressure
PaO_2_: Arterial Oxygen Partial Pressure
pH: Pondus Hydrogenii
qRT-PCR: Quantitative real-time PCR
SD: Standard Deviation
